# Variations of the Functional Brain Network Efficiency in a Young Clinical Sample within the Autism Spectrum: A fNIRS Investigation

**DOI:** 10.3389/fphys.2018.00067

**Published:** 2018-02-05

**Authors:** Yanwei Li, Dongchuan Yu

**Affiliations:** ^1^College of Preschool Education, Nanjing Xiaozhuang University, Nanjing, China; ^2^Key Laboratory of Child Development and Learning Science of Ministry of Education, School of Biological Sciences and Medical Engineering, Southeast University, Nanjing, China

**Keywords:** autism spectrum disorder, individual difference, early brain development, fNIRS, complex functional brain network

## Abstract

Autism is a neurodevelopmental disorder with dimensional behavioral symptoms and various damages in the structural and functional brain. Previous neuroimaging studies focused on exploring the differences of brain development between individuals with and without autism spectrum disorders (ASD). However, few of them have attempted to investigate the individual differences of the brain features among subjects within the Autism spectrum. Our main goal was to explore the individual differences of neurodevelopment in young children with Autism by testing for the association between the functional network efficiency and levels of autistic behaviors, as well as the association between the functional network efficiency and age. Forty-six children with Autism (ages 2.0–8.9 years old) participated in the current study, with levels of autistic behaviors evaluated by their parents. The network efficiency (global and local network efficiency) were obtained from the functional networks based on the oxy-, deoxy-, and total-Hemoglobin series, respectively. Results indicated that the network efficiency decreased with age in young children with Autism in the deoxy- and total-Hemoglobin-based-networks, and children with a relatively higher level of autistic behaviors showed decreased network efficiency in the oxy-hemoglobin-based network. Results suggest individual differences of brain development in young children within the Autism spectrum, providing new insights into the psychopathology of ASD.

## Introduction

Autism Spectrum Disorders (ASD), a life-long neurodevelopmental disorder, is characterized by impaired social communication and social interaction, and restrictive interests and repetitive behaviors (APA, 2013). As reviewed in Elsabbagh et al. (2012), researchers estimated that the global prevalence of ASD is 62/10,000. Moreover, they found that the prevalence of ASD has increased over time. For example, in US, data reports from Autism and Developmental Disabilities Monitoring Network suggest the estimated prevalence of eight-year-old children with ASD in 2012 was more than twice that of 2000 (CDC, 2014). Although the cause of the increasing ASD rates is unclear, investigators believe that it is associated with the broader diagnostic criteria, early diagnosis at very young age, availability of services, and even greater public awareness (Elsabbagh et al., 2012; Rice et al., 2012; Hansen et al., 2015). Thus, ASD is a disorder of increasing attention from not only the public, education system, and medical care, but also from the scientific community.

The presentation of impaired behaviors in ASDs varies in range and severity of symptoms. The variability within this neurodevelopmental disorder creates an extremely heterogeneous group that differs in course and response to treatment (Ingersoll et al., 2001; Farley et al., 2009). For example, ASD varies in the severity of symptoms and the acquisition of cognitive and social functioning. Researchers found that some ASDs with mild symptoms were capable of leading independent lives, while others with severe symptoms required moderate or extensive assistance from their families and local service authorities (Farley et al., 2009). Moreover, Evidence suggests that ASDs would benefit differently from the same behavioral intervention programs due to their great individual differences. Using a prospective study design, Sherer and Schreibman (2005) indicated that young children exhibited different levels of positive changes after a period of an approximately 190 h intervention. Various positive outcome changes were evidenced for children with best favorable behaviors (i.e., a higher level of social engagement, intellectual ability and linguistic skills) other than those with the most unfavorable behaviors (i.e., a lower level of social engagement, intellectual ability and linguistic skills). Thus, for a wide range of individuals with Autism, a comprehensive understanding of individual difference would be necessary. Only under such circumstances would individuals with Autism who experience severe behavioral and cognitive challenges get appropriate, specified, and effective behavioral intervention programs (Cicchetti and Curtis, 2006; Dawson et al., 2008).

Autism Spectrum Disorder has been proved to be related to a lot of brain abnormalities (i.e., damages in the structural and functional brain). As reviewed in Stigler et al. (2011) and Ulay and Ertugrul (2009), ASD was connected to the enlarged head circumference, the increased total cerebral volume, and rapid brain overgrowth during the first few years of life. It was also connected to aberrant activation in the temporal lobes and amygdala while investigating the neural circuitry of language and social cognition. Moreover, neural network deficits in ASD were evidenced based on the default-mode network studies, with decreased functional connectivity in the frontal and angular gyrus (Kennedy and Courchesne, 2008). However, due to the rigorous measurement environment required by the good neuroimaging data quality (Philip et al., 2012), few studies explored the neurological development of young ASDs (younger than 8 years old). Recently, the emerging application of a new non-invasive neuroimaging approach, functional near-infrared spectroscopy (fNIRS), has proven to be useful in exploring the brain development even in young individuals with psychopathology, especially for young children with ASD. It is not sensitive to head movement and can be used in the open environment measurement, providing more opportunities to investigate the early brain development of young ASDs. For example, using fNIRS, lower levels of long-range connections between the bilateral prefrontal cortexes were found in young children (aged 4.8–8 years old) with ASD in our previous study (Li and Yu, 2016), supporting the under-connectivity hypothesis (Just et al., 2004). Using a longitudinal design, Keehn et al. (2013) found that infants at high risk for ASD exhibited aberrant functional brain connectivity in the frontal-temporal area when compared with age-matched infants at low risk for ASD by fNIRS, with increased connectivity at 3 months but decreased connectivity at 12 months. Such evidence suggests a dynamic changing model of brain abnormalities in Autism. It broadens our understanding of the neurodevelopment of young children with ASD and suggests potential neuroimaging biomarkers for the early diagnosis of Autism and the evaluation of its intervention effect.

Recently, researchers tried to reveal the neuroimaging features of psychological disorders, aiming to find some neural biomarkers and to assist in the precise diagnosis of clinical samples. Due to a lack of detailed illness narratives of some patients, such objective neuroimaging-based brain biomarkers would help more when diagnosing individuals at a lower level of interaction skills (i.e., young clinical samples). Thus, researchers started to explore evidence for the relationships between structural and functional neuroimaging features and typical behaviors in young clinical samples (i.e., children with irritability, Attention Deficit Hyperactivity Disorder, ASD and so on). For example, Grabell et al. (2018) investigated the relationship between prefrontal activation and 3- to 7- year-old children's levels of irritability during a frustration-related task by fNIRS. They found there was an inverted-U function between them in the left PFC, and the inverted-U apex of irritability score was approximate to the 1.5 standard deviations clinical cutoff score defined by a large scale behavioral study conducted by Wakschlag et al. (2015). Another study (Gu et al., 2017) conducted on children with Attention Deficit Hyperactivity Disorder (ADHD) by fNIRS indicated that the permutation entropy value, a measure representing the complexity of fNIRS, in the dorsolateral cortex, was considerably larger than that in the control groups. Furthermore, the higher level of permutation entropy value of right dorsolateral prefrontal cortex was related to severe ADHD symptoms. Such evidence supported that neuroimaging features were able to be used to represent the typical symptoms of young clinical children and be regarded as promising biomarkers. Similar studies have also been conducted in young children with ASD by structural MRI. Munson et al. (2006) found that, for young children (3–4 years old) with ASD, larger right amygdala volume was related to more severe social and communication deficits. Therefore, it is valuable for researchers to uncover the relationship between brain abnormalities and individual difference in the severity of symptoms in young children with ASD. Since ASD is a disorder outbursts at an early age (several months old) and exhibits both behavioral and brain impairments in the subsequent development, it's essential to explore brain abnormalities from the developmental perspective as well. A previous study found that, compared with the age-matched typically developing children (7.5–18.5 years old), children with ASD failed to gain the age-related amygdala increase (Schumann et al., 2004). All of the above inspired us to reveal the variations of brain abnormalities in young ASDs from the perspective of age and severity of their typically autistic behaviors.

The concepts of complex functional brain network have been combined with the fNIRS technique in recent years. Compared with fMRI, fNIRS has relatively higher temporal resolution, which makes it possible to segregate the cardiac and respiratory noise from the neural signals (Tong et al., 2011). It could provide not only oxy-hemoglobin signals but also deoxy- and total-hemoglobin signals of the cerebral blood flow, describing a more comprehensive picture of the topological networks (Duan et al., 2012). Moreover, Niu et al. (2013) have proved that small-world analysis is reliable in fNIRS investigations. Thus, fNIRS could be regarded as a good choice to construct functional brain networks and be applied to network topology analysis of young children with ASD. As described before, researchers found that infants at high risk for ASD exhibited an aberrant pattern of frontal-temporal functional brain connectivity by fNIRS (Keehn et al., 2013). Researchers also found that, compared with typically developing children, ASDs displayed reduced functional connectivity between the bilateral temporal cortices during the resting state (Zhu et al., 2014) and lower levels of functional links between the bilateral prefrontal lobes while watching cartoon (Li and Yu, 2016). However, most of the studies focused only on local functional connectivity rather than global network features, such as the characteristic values defined by Latora and Marchiori (2001) (i.e., small-worldness, network efficiency and so on). In addition, few results were consistent and it was difficult to comprehensively interpret the whole picture. For example, opposed to the under-connectivity hypothesis (Just et al., 2004), Kikuchi et al. (2013) found that children with ASD aged 3–7 years old exhibited higher inter-hemisphere functional connectivity between the bilateral anterior prefrontal cortexes than typically developing children (Kikuchi et al., 2013). Recently, researchers succeeded in using the global and local network efficiency as characteristic features to discriminate young children with and without ASD, suggesting network efficiency a potential feature in describing the aberrant functional network (Li and Yu, 2016). Further explorations should aim to investigate whether network efficiency is a good index in representing the variations of brain abnormalities in Autism.

Our current study has two main goals, exploring the individual difference of global and local network efficiency in young children with ASD from the perspective of age and then from the perspective of the severity of typical autistic behaviors. Using fNIRS, we tested forty-six 2–8-year-old children with ASD while they were watching a cartoon. We hypothesized the global and local network efficiency would be associated with age and severity of typical autistic behaviors, which means network efficiency would be a potential biomarker for ASD. We expected children with higher levels of autistic behaviors would exhibit a lower level of network efficiency based on the weak network efficiency deficits in young children with ASD (Li and Yu, 2016) and under-connectivity hypothesis (Just et al., 2004). Early years are the most important stage for the construction of neural wiring supporting the functioning of cognition and emotion, suggesting an increase of the information exchange efficiency with age in healthy children. As opposed to this pattern in healthy children, we hypothesized young ASDs would fail to gain the same increase.

## Materials and methods

### Participants

Fifty-six children with ASD participated in the current study. All children were recruited from a local special school (*NANJING MINGXIN Intelligence-Promoting School, NJMXIPS*). All of them received a diagnosis of autism at a local hospital (NANJING Brain Hospital) according to the criteria for autism in the fourth edition of *Diagnostic and Statistical Manual (DSM-IV)* no longer than two years ago. Based on their daily behaviors and communication with their teachers, they were identified as high-functioning autism by their teachers, indicating they exhibited an approximately normal range of intelligence. Besides, those children had never been diagnosed with the comorbidity of ADHD or any other psychiatric diagnosis. Parents of all participants signed the written informed consent for this study, which was approved by the Ethics Committee of the Southeast University.

Seven of those children were excluded from data analysis because of the failure of fNIRS data collection. Another three of the remaining participants failed to complete the behavior investigation as we mentioned below. Thus, the final clinical dataset was 46 children with ASD (36 boys; age range is 2.0–8.9 years old; mean age = 5.0 years; *SD* of age = 1.7).

### Parent-rated autism behaviors

The Autism Behavior Checklist (ABC) (Yirmiya et al., 1994), a parental report questionnaire, was used to evaluate the level of autistic behaviors. It comprised 57 items rated on a 2-point scale (yes or no), describing Autism related behaviors in five areas which are sensory, relating, body and object use, language, and social and self-help skills. A weighted score (1–4) was assigned for each of the items, depending on its association with the five investigated areas. Then the weighted score for each item was added up in each area and the whole questionnaire so as to represent the subscale score and total scale-score of ABC. The total scale-score of ABC would be used as the evaluation for the severity level of autistic behaviors.

### Experimental protocol

In agreement with our previous research, children were requested to watch a Chinese cartoon while their brain was monitored by fNIRS. The whole scanning process was about 10 min. The following strategies were adopted to ensure the success of fNIRS data collection:

- A month before the formal experiment, children were required to wear a cap which was similar to the electrode one used in the formal experiment for about 10 min every day, aiming to ensure their willingness of wearing the electrode cap on the experiment day.
- During the scanning process, children were asked to keep quiet while they were watching the first episode of a popular Chinese cartoon named “BEAR.” At the same time, their parents or teachers were told to hold the child comfortably so as to make sure he/she would feel safe during the process.
- Children's large body movements or unexpected behaviors were recorded simultaneously by a camera with the permission of their parents, which were used to specify the corrupted data points afterwards.

### fNIRS data acquisition and analysis

#### Data acquisition

**Figure 1:**
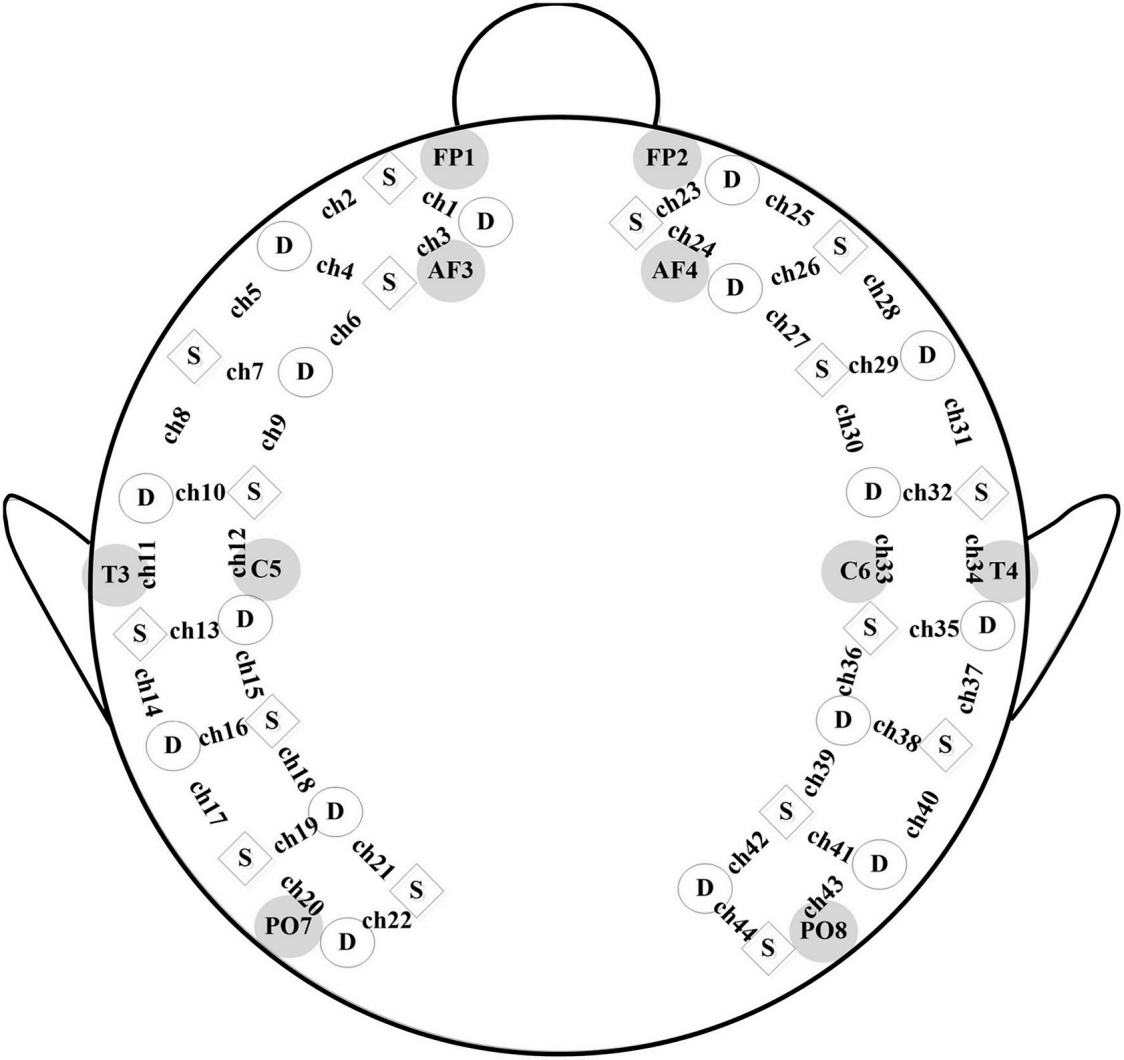
fNIRS probe. A schematic of the customized fNIRS cap layout design including the reference of international 10/20 coordinates, with squares representing source emitters and circles representing detectors.

As previously described by Li and Yu (2016), a real-time fNIRS system, *LABNIRS* (Shimadzu Corporation, Kyoto, Japan) was used to collect the optical imaging data at the Laboratory of Cognitive Neuroscience in Southeast University. The fNIRS probe comprised 16 light-source positions and 16 detectors. Each light source could generate three wavelengths (780, 805, and 830 nm) of near-infrared light, which were received by the nearest detector after traveling through the outer layer of the brain. The connection between each pair of source and detector represented a 3 cm measurement channel. The hemoglobin (oxy-, deoxy-, and total-hemoglobin) levels were recorded by estimating the absorption of the three wavelengths of near-infrared light for each measurement channel using the modified Beer-Lambert Law (Cope and Delpy, 1988). A customized electrode cap, consisting of 44 measurement channels, was made to hold the position of each sensor as marked in Figure 1. The positioning of each sensor was determined according to the international 10–20 system. The frontest sensors were placed around FP1 and FP2, the backmost sensors were placed around PO7 and PO8, the leftmost sensors were placed around T3, and the rightmost sensors were placed around T4. The scanning rate was 27 ms based on the montage of sources and detectors. The cap was put on each child's head by aligning its central to the position of Cz, the front middle to FpZ, and the leftmost and rightmost sensors next to T3 and T4, respectively. The hair under each sensor was manually parted so as to improve the signal-to-noise ratio of each measurement channel. It took two experimenters 5–10 min to complete the whole preparatory process.

#### Data preprocessing

**Figure 2:**
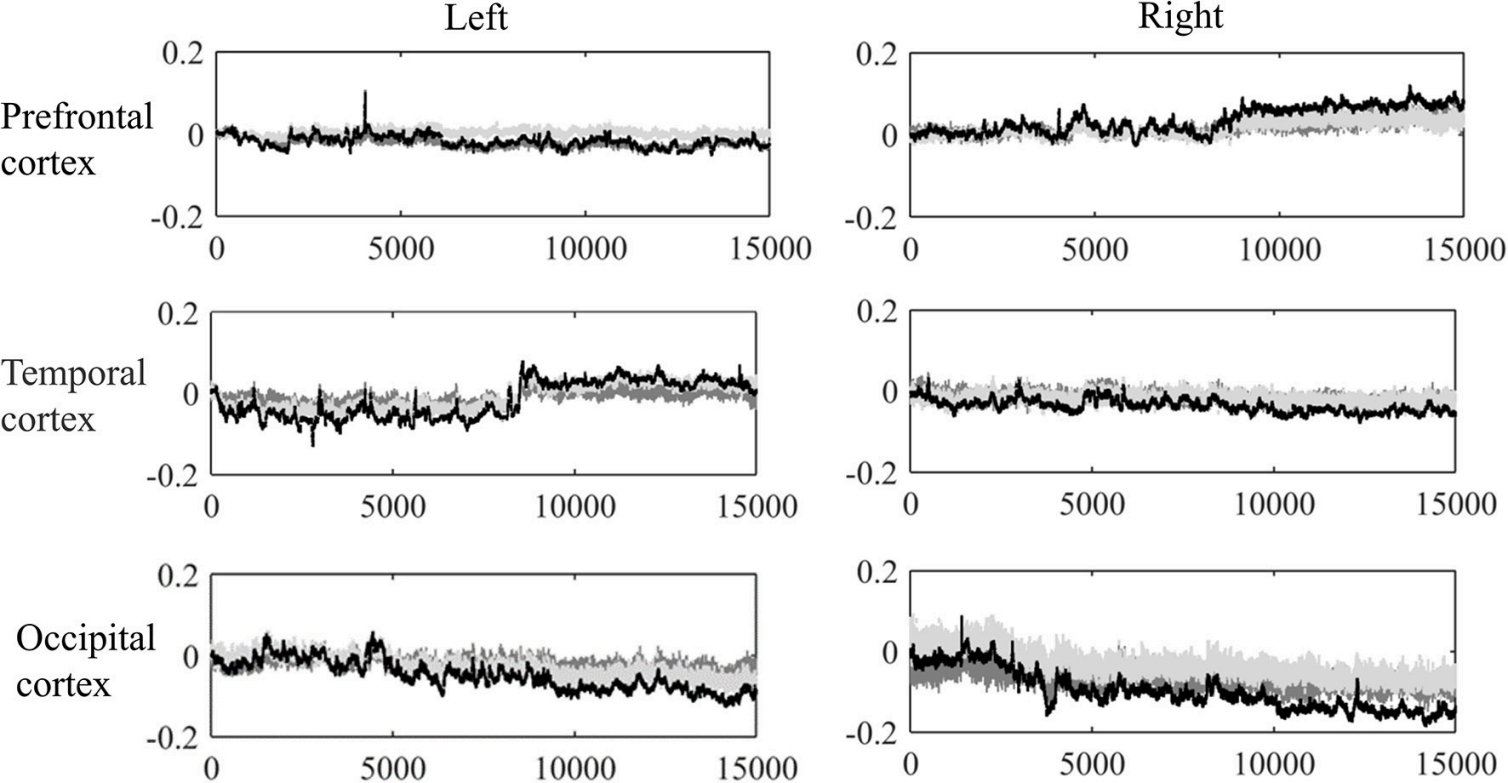
Grand average of the dynamic hemoglobin concentration changes for one representative channel in each lobe.

Corrupted data points were excluded according to the camera recording, such as those caused by large head movements or unexpected behaviors. Moreover, a visual inspection (Nakano et al., 2009; Niu et al., 2012) was conducted to remove the obvious sharp changes in the time series of hemoglobin changes. Thus, 6.9% of the original data points were ruled out, and the mean time-length of the data was 10.24 min. Figure 2 shows the grand average of the hemodynamic response for one representative channel in each lobe based on the first 15,000 sample points (approximate 7 min). At last, a band-pass filtering (0.009–0.08 Hz) was conducted on the raw hemoglobin concentration data (Zhang et al., 2009; Cui et al., 2011; Sasai et al., 2011), so as to remove the effects of low-frequency drift and high-frequency neurophysiological noise.

#### Network construction

Nodes and edges are the key elements in complex network analysis (Bartsch et al., 2015; Ivanov et al., 2016). In the current study, nodes are the measurement channels between each pair of source-detector, and edges are the connections among nodes computed by Pearson correlation. Thus, for the current fNIRS probe, each subject got 44 nodes and a 44 × 44 correlation matrix with 946 effective links. Subsequently, the correlation matrix was converted into an undirected binary graph (*G*) by employing a pre-configured correlation threshold δ. A study conducted by our group (Li and Yu, 2016) suggest that the degree of discrimination between ASD and non-ASD would decrease dramatically after a threshold larger than 0.6. Thus, the high end of the threshold range was set at 0.6. Meanwhile, we set the low end of the threshold at 0.2 based on the evidence that a correlation efficient of 0.1 would only be regarded as a small interpret effect size (Cohen, 1988). Thus, the threshold range was set from 0.2 to 0.6, with an interval = 0.1. Edges with an absolute correlation coefficient larger than δ were defined as 1 (with connectivity). Otherwise, were defined as 0 (without connectivity).

The network metrics were computed by the GRETNA package (Wang et al., 2015). Global efficiency (*E*_*glob*_) is used to describe the capability of information transmission at a global level, which equals to the averaged reciprocal of shortest path length between nodes; Local efficiency (*E*_*loc*_) is used to illustrate the ability of information exchange at a local level, which equals to the averaged efficiency of the subgraph (Latora and Marchiori, 2001). For each δ ϵ {0.2, 0.3, 0.4, 0.5, 0.6}, we could obtain a pair of *E*_*glob*_ and *E*_*loc*_ for each topological organization for each participant. Algorithm equations of network efficiency (Latora and Marchiori, 2001; Wang et al., 2009) are as follows:

$$\begin{array}{l} E_{glob}=\frac{1}{N\left(N-1\right)}\underset{i\neq j\in G}{\sum }\frac{1}{L_{i,j}} \end{array}$$

where *N* is the total number of nodes in a network (44 in the current study), and *L*_*i,j*_ is the shortest path between a node *i* and another node j.

$$\begin{array}{l} E_{loc}=\frac{1}{N}\underset{i\in G}{\sum }E\left(G_{i}\right) \end{array}$$

where *G*_*i*_ is the local subgraph and *i* ∉ *G*_*i*_, meaning *G*_*i*_ consisting of only the immediate neighbors of a node *i* but not itself.

### Analysis strategy

Children recruited in the current study were from a wide range of age and exhibited different levels of autistic behaviors. Thus, we firstly tested for the association between age and the total scale-scores of ABC to see if there was sampling bias. Secondly, using correlation, we tested if network efficiency is related to age and levels of autistic behaviors. We hypothesized that the *r* values were independent of each other because the topologies were distinct for each δ ϵ {0.2, 0.3, 0.4, 0.5, 0.6}, in the oxy-, deoxy-, and total-Hb-based networks, respectively. Thus, no multiple comparison correction approaches were used in the current study. It should be noted that if age was related to the levels of autistic behaviors, partial correlation analysis would be conducted in the second step instead of the bivariate correlation analysis.

## Results

### Levels of autistic behaviors and its association with age

Parents reported a range of levels of autistic behaviors (*Mean* = 61.33, *SD* = 21.79, *Range* 33–119). Using the bivariate correlation analysis, we found that age was significantly positively correlated with levels of autistic behaviors [*r*_(46)_ = 0.51, *p* < 0.001]. Thus, partial correlation was used in the following analysis. Table 1 summarizes the means and standard deviations of each subscale in the current sample. A further bivariate analysis suggests that age was also related to the scores of all subscales in ABC (*r*-values ranged from 0.306 to 0.416, *p* < 0.05).

**Table 1:** Means and standard deviations of ABC subscales.

	**Mean**	***SD***	**Range**
Sensory	8.59	4.89	0–20
Relating	15.78	6.43	2–31
Body and object use	11.65	8.21	0–31
Language	14.26	6.14	5–30
Social and self-help skills	11.04	4.32	4–20

### Association between network efficiency and age

Using partial correlation, we tested whether global and local network efficiency in the oxy-, deoxy-, and total-Hb-based-network topology was associated with age, respectively, when controlling for levels of autistic behaviors.

In the oxy-Hb-based-network, age was not associated with the global or local network efficiency, for δ ϵ {0.2, 0.3, 0.4, 0.5, 0.6} (*r*-values for *E*_glob_ ranged from −0.198 to −0.124, *p* > 0.05; *r*-values for *E*_loc_ ranged from 0.081 to −0.025, *p* > 0.05).

In the deoxy-Hb-based-network, age was significantly negatively related to global network efficiency for δ ϵ {0.4, 0.5} (*r*-values ranged from −0.335 to −0.324, *p* < 0.05) and local network efficiency for δ = 0.4 (*r* = −0.322, *p* < 0.05). Age was also marginally negatively related to global network efficiency for δ = 0.6 (*r* = −0.293, *p* = 0.051) and local network efficiency for δ ϵ {0.3, 0.5} (*r*-values ranged from −0.282 to −0.251, 0.05 < *p* < 0.1).

In the total-Hb-based-network, age was significantly negatively related to global network efficiency for δ ϵ {0.2, 0.3, 0.4, 0.5} (*r*-values ranged from −0.413 to −0.305, *p* < 0.05) and local network efficiency for δ = 0.3 (*r* = −0.352, *p* < 0.05). Age was also marginally negatively related to global network efficiency for δ = 0.6 (*r* = −0.255, *p* = 0.090) and local network efficiency for δ = 0.4 (*r* = −0.277, *p* = 0.066).

**Figure 3:**
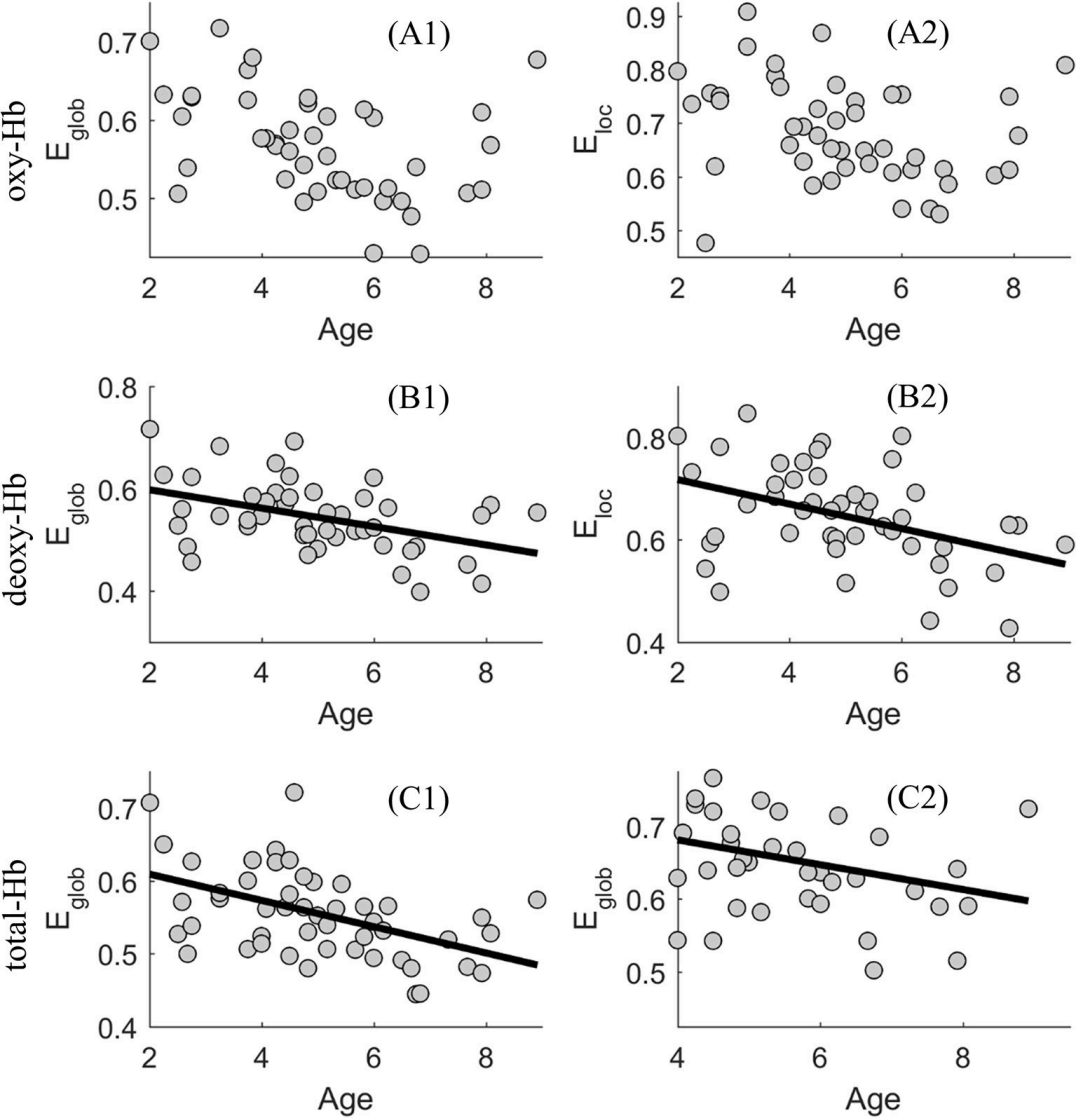
Scatter plot showing the association between age and the global (*E*_*glob*_) and local (*E*_*loc*_) network efficiency in the oxy-(**A1** and **A2**), deoxy-(**B1** and **B2**), and total-Hb-based (**C1** and **C2**) networks.

According to the above results, significant associations between age and the network efficiency were especially evident when δ = 0.4, in the deoxy- and total-Hb-based-networks. Thus, using a scatter plot, Figure 3 shows the associations between age and the global and local network efficiency in the oxy-, deoxy-, and total-Hb-based networks when δ = 0.4.

### Association between network efficiency and levels of autistic behaviors

Using partial correlation, we tested whether global and local network efficiency in the oxy-, deoxy-, and total-Hb-based-network topology was associated with levels of autistic behaviors, respectively, when controlling for age.

In the oxy-Hb-based-network, the level of autistic behaviors was significantly negatively associated with the global network efficiency, for δ ϵ {0.2, 0.3, 0.4, 0.5} (*r*-values ranged from −0.432 to −0.395, *p* < 0.01); it was also significantly negatively associated with the local network efficiency, for δ ϵ {0.2, 0.3, 0.4, 0.5, 0.6} (*r*-values ranged from −0.393 to −0.324, *p* < 0.05).

In the deoxy-, and total-Hb-based-network, levels of autistic behaviors was not related to the global or local network efficiency for δ ϵ {0.2, 0.3, 0.4, 0.5, 0.6} (*r*-values for *E*_glob_ ranged from −0.212 to −0.054, *p* > 0.05; *r*-values for *E*_loc_ ranged from 0–0.239 to −0.058, *p* > 0.05).

**Figure 4:**
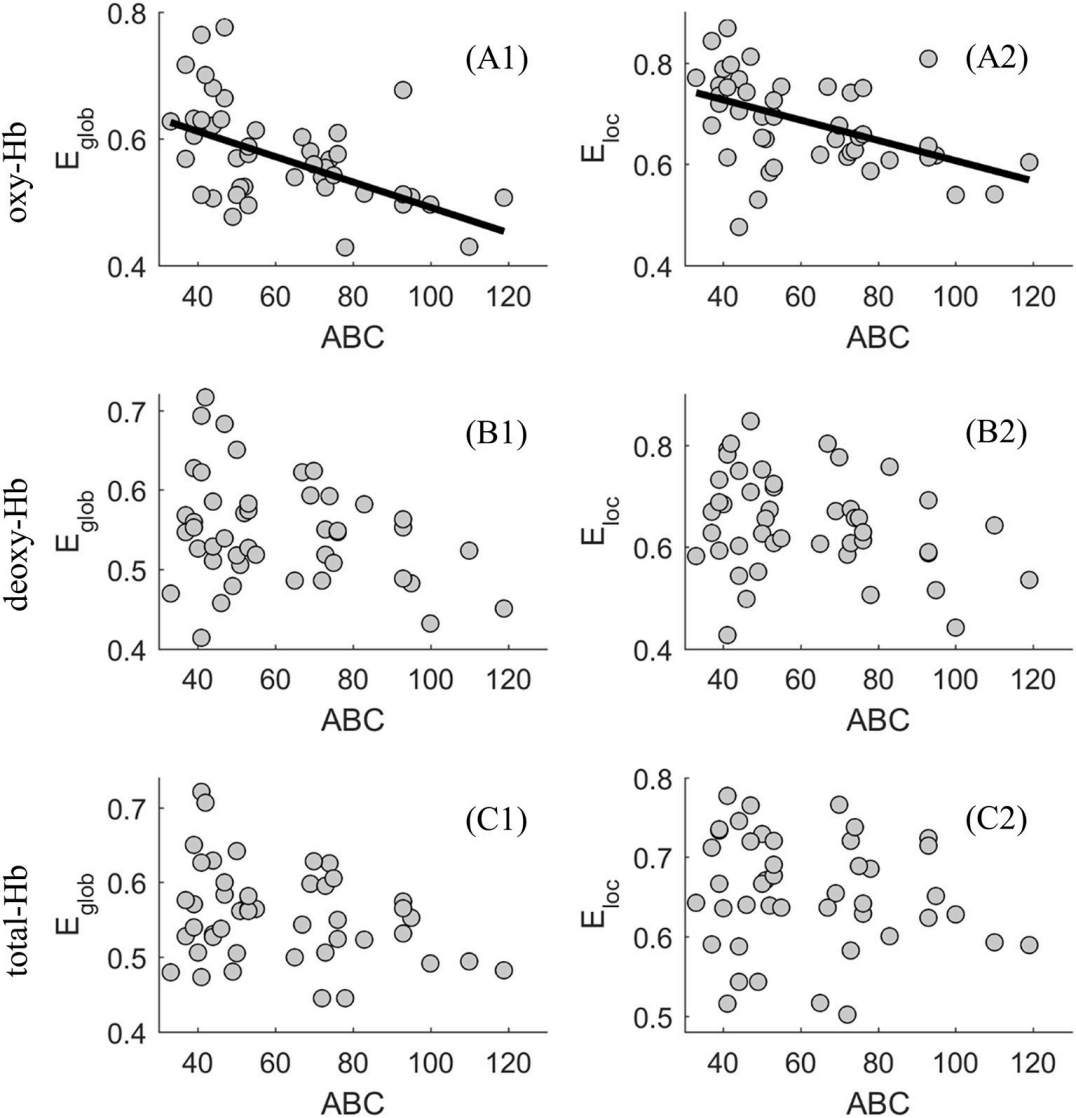
Scatter plot showing the association between levels of autistic behaviors and the global (*E*_*glob*_) and local (*E*_*loc*_) network efficiency in the oxy-(**A1** and **A2**), deoxy-(**B1** and **B2**), and total-Hb-based (**C1** and **C2**) networks.

Thus, for a wide range of threshold values (δ ϵ {0.2, 0.3, 0.4, 0.5, 0.6}, significant associations between levels of autistic behaviors and the network efficiency were found in the oxy-Hb-based-network. Using a scatter plot, Figure 4 provides an example (δ = 0.4) showing the associations between levels of autistic behaviors and the global and local network efficiency in the oxy-, deoxy- and total-Hb-based networks.

**Figure 5:**
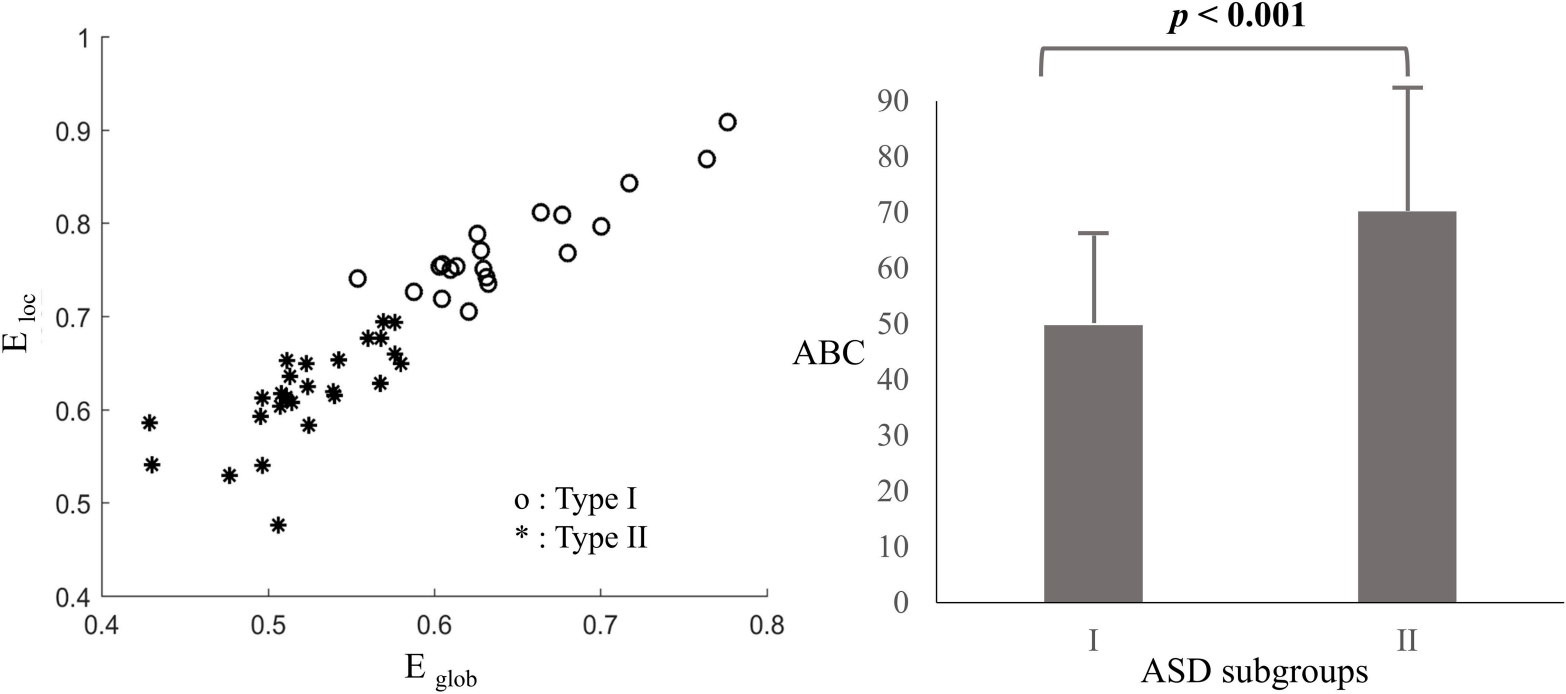
Clustering results based on the oxy-Hb-based networks **(Left)** and means and standard deviations (*SD*) of the total ABC scores for Type I and II ASD **(Right)**, with error bars representing the SDs.

Clustering and classification techniques have been widely used in the grouping of similar functional networks, such as K-means clustering analyses (Yu, 2013; Li and Yu, 2016), consensus approach (Rasero et al., 2017), and so on. Based on the association between network efficiency and levels of autistic behaviors, the K-means clustering analysis was performed to divide the clinical subjects into two groups when δ = 0.4, with the global and local network efficiency as feature parameters. Clusters of a population with a higher level of network efficiency (Type I ASD, *n* = 20) were compared with clusters of a population with a lower level of network efficiency (Type II ASD, *n* = 26). The results, shown in Figure 5, clearly implicated the clustering shape of the two types of ASD. And according to the Figure 5, the mean of total ABC scores in Type I ASD was significantly lower than that in Type II ASD (*t* = 3.522, *p* < 0.001).

## Discussion

In the present study, we found that ASD with older age was related to more severe autistic symptoms, suggesting a sampling bias which was difficult to avoid during the recruitment of subjects. Thus, partial correlation was used when exploring the association between the functional brain network efficiency and age or severity of autistic symptoms, with the effect of severity of autistic symptoms or age removed, respectively. The partial correlation analysis revealed that significant associations between age and the network efficiency were especially evident when δ = 0.4, in the deoxy- and total-Hb-based-networks, indicating the network efficiency would decrease with age in young ASDs. In addition, when controlling for the effect of age, significant associations between levels of autistic behaviors and the network efficiency were found in the oxy-Hb-based-network for a wide range of threshold values (δ ϵ {0.2, 0.3, 0.4, 0.5, 0.6}). ASDs with a relatively higher level of autistic behaviors showed decreased functional global and local network efficiency based on the oxy-hemoglobin constructed topological figure.

As described before, researchers have already proved that parent- or self- reported behavioral measures were related to the structural brain measures and functional brain measures during some special mental states (Munson et al., 2006; Gu et al., 2017; Li et al., 2017; Grabell et al., 2018). Such results indicated that brain measures might be effective indexes in describing the individual difference. Consistent with those findings, the current study proved that the functional network efficiency of the oxy-hemoglobin-based topological network was a good biological index in describing the individual differences of autistic behavior for young ASDs. We found there was a negative association between the functional brain network features and parent-rated autistic behaviors. Being more specific, based on the oxy-hemoglobin constructed topological network, Type I ASD with a relatively higher level of network efficiency showed decreased lower levels of autistic behaviors when compared with Type II ASD who exhibited a relatively lower level of network efficiency. The results suggest that young ASDs with severe autistic behaviors would exhibit lower information exchange efficiency. Such a result was supported by our previous finding that young ASDs would exhibit reduced global and local network efficiency when compared with typically developing children (Li and Yu, 2016). Meanwhile, the result was also supported by previous studies (Lewis et al., 2013, 2014) conducted on infants and adults with ASD while investigating their structural brain networks features. Lewis et al. (2013, 2014) found infants and adults with ASD would exhibit weak global and local network efficiency in multiple regions of interest in the whole brain, suggesting information exchanging deficits from the perspective of the structural brain as well. Here, the global and local network efficiency was proved to be an effective index in characterizing the individual difference of ASD psychopathology. Future studies should focus on using their association to define the clinical cutoff score of the behavioral measure, which should be very close to that defined by a large-scale study using standard deviations. If so, the global and local network efficiency measures could be regarded as potential biomarkers for discriminating between clinical population and healthy controls. A representative work conducted by Grabell et al. (2018) suggested a very useful example in determining the behavioral cutoff of clinical and non-clinical individuals by exploring the non-linear relationship between brain and behavioral measures.

Autism is a neurodevelopment disorder with very early onset of clinical symptoms (i.e., language delay, emotion dysregulation, and social avoidance) (Wetherby et al., 2004; Landa and Garrettmayer, 2006) and life-long behavioral and brain impairments in the subsequent development. For typically developing individuals, remarkable gains in social and cognitive learning emerge during the early childhood, along with evident brain development. Thus, it's essential to explore the brain impairments of ASDs from the developmental perspective, especially for the period of early years in life. However, few of previous researchers investigated the developmental features of brain during this period, which is the most important stage for the construction of neural wiring supporting the process of cognitive and social functioning. Here, the current study found a significant negative association between age and the network efficiency in the deoxy- and total-Hb-based-networks, which was especially evident when δ = 0.4. The results indicate the network efficiency would decrease with age in young ASDs. Such results supported our hypothesis. Moreover, the results acted in cooperation with previous work on the structural brain in ASDs. Courchesne et al. (2007) came up with a model describing the early brain development in Autism. They suggested a phase of overgrowth at the beginning of life and a phase of slowing or arrest growth during early childhood. For example, Schumann and colleagues found that compared with the age-matched typically developing children (7.5–18.5 years old), children with ASD failed to gain an age-related amygdala increase. Another explanation for the decrease of network efficiency in the early years of ASD was that major event of neural circuit was disrupted by the excessive number of neurons (Courchesne et al., 2007), resulting reduced long-distance functional connections (Just et al., 2004) which in turn hindered the information exchange efficiency in the whole brain. In all, the current study is among the first to explore the functional network features of ASD from the developmental perspective, giving a hint on how functional brain develops in young ASDs.

Inconsistent results were reported among the three kinds of functional networks (i.e., oxy-, deoxy-, and total-Hb-based-network). For example, age was negatively related to the global and local network efficiency in both the deoxy- and total-Hb-based functional network, but not that in the oxy-Hb-based functional network. While, levels of autistic behaviors were proved to be negatively related to the global and local network efficiency in the oxy-Hb-based functional network, but not that in the deoxy- or total-Hb-based functional network. Such results supported previous findings (Gervain et al., 2011; Sasai et al., 2011; Perlman et al., 2014; Li and Yu, 2016) on the varying conclusion among the three kinds of hemoglobin signals, which would be helpful to characterize their properties.

As opposed to previous work on Autism Spectrum Disorder (Pelphrey et al., 2005; Munson et al., 2006), the current study lacks an ASD diagnostic confirmation for children who visited our lab. This is because there is limited access to the golden diagnostic tools of ADI-R or ADOS in China (Liu et al., 2016). Another limitation of the current study may be the sampling bias which was difficult to avoid during the recruitment of subjects. The current study found that severity of ASD increased with age. Given the relatively small sample size and limited population studied, we hypothesized it could be related to the clinical diagnosis and implementation of interventions for children with ASD. Those children would exhibit severer symptoms if they couldn't receive timely diagnosis and help form appropriate intervention programs. In order to reduce the adverse impact of sampling bias in the current study, partial correlation was conducted in the data analysis. The final limitation is about fNIRS methodology. This imaging technique could only monitor the hemoglobin changes in the outer cortex, and the limited number of emitters and detectors could only construct a probe covering the bilateral prefrontal, temporal, and occipital areas. Hence, the topological network in the current study didn't contain any deep brain areas (i.e., anterior insula and anterior cingulate cortex) and the outer layer of the parietal cortex. However, the current study is among the first to explore the association between the functional network efficiency features and individual differences in young children with Autism Spectrum Disorder by fNIRS.

In conclusion, we found significant partial correlations between age and the network efficiency, between levels of autistic behaviors and the network efficiency, when controlling for the effect of levels of autistic behaviors or age, respectively. The results indicate the network efficiency would increase with age in young ASDs. In addition, the results indicate that ASDs with a relatively higher level of autistic behaviors exhibited reduced functional global and local network efficiency. The current study sets the scene for exciting future research exploring the neurodevelopmental features of young children with ASD, and suggesting a potential biomarker to discriminating ASDs from healthy controls.

## Author contributions

DY and YL: Conceived and designed the experiments, analyzed the data, contributed reagents, materials, analysis tools, wrote the paper; YL: Performed the experiments.

### Conflict of interest statement

The authors declare that the research was conducted in the absence of any commercial or financial relationships that could be construed as a potential conflict of interest.
